# Supplementary material for: Causes and consequences of sex-chromosome turnovers in Diptera
Source: Evol Lett. 2026 Mar 12;10(3):253–68. doi: 10.1093/evlett/qrag003 (PMC13231274; doi:10.1093/evlett/qrag003)
Supplement: qrag003_Supplemental_Files [file qrag003_supplemental_files.zip › Supplementary_Datasets.zip!/Supplementary_Datasets/Dataset_2_Graphical_representation_outgroups/Stratiomydae_Beris_chalybata_chromosomes.pdf]

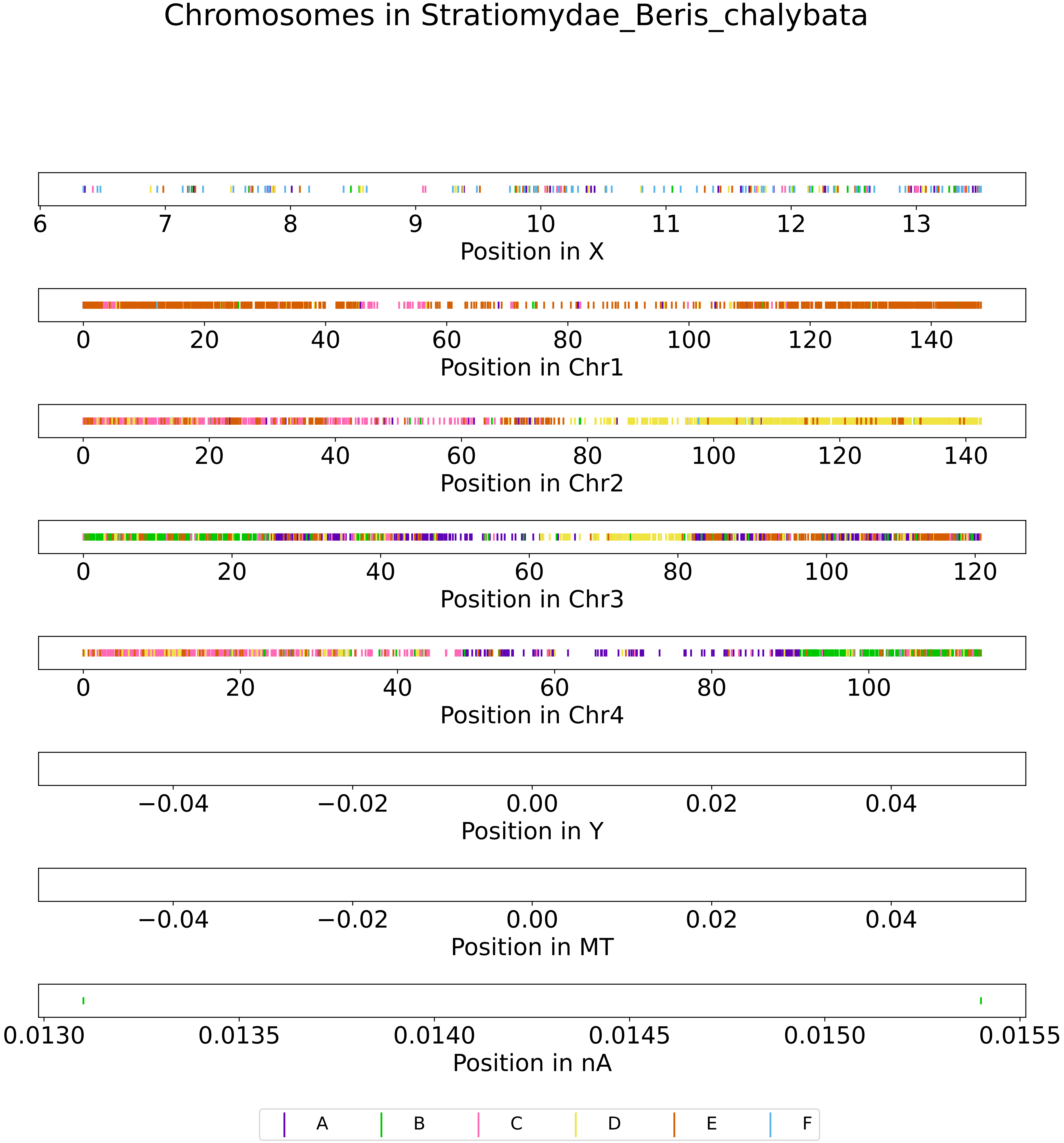

Chromosomes in Stratiomydae_Beris_chalybata
6
7
8
9
10
11
12
13
Position in X
0
20
40
60
80
100
120
140
Position in Chr1
0
20
40
60
80
100
120
140
Position in Chr2
0
20
40
60
80
100
120
Position in Chr3
0
20
40
60
80
100
Position in Chr4
−0.04
−0.02
0.00
0.02
0.04
Position in Y
−0.04
−0.02
0.00
0.02
0.04
Position in MT
0.0130
0.0135
0.0140
0.0145
0.0150
0.0155
Position in nA
A
B
C
D
E
F
